# Transmission-Mode Ultrasound for Monitoring the Instantaneous Elastic Modulus of the Achilles Tendon During Unilateral Submaximal Vertical Hopping

**DOI:** 10.3389/fphys.2020.567641

**Published:** 2020-12-03

**Authors:** Scott C. Wearing, Larissa Kuhn, Torsten Pohl, Thomas Horstmann, Torsten Brauner

**Affiliations:** ^1^Institute of Health and Biomedical Innovation, Queensland University of Technology, Brisbane, QLD, Australia; ^2^Faculty of Sports and Health Sciences, Technical University of Munich, Munich, Germany; ^3^Department of Sport Science, German University of Health and Sport, Ismaning, Germany

**Keywords:** speed of sound, tendon, stretch-shortening cycle, muscle, biomechanics, elastic modulus, elasticity

## Abstract

Submaximal vertical hopping capitalizes on the strain energy storage-recovery mechanism associated with the stretch-shortening cycle and is emerging as an important component of progressive rehabilitation protocols in Achilles tendon injury and a determinant of readiness to return to sport. This study explored the reliability of transmission mode ultrasound in quantifying the instantaneous modulus of elasticity of human Achilles tendon during repetitive submaximal hopping. A custom-built ultrasound transmission device, consisting of a 1 MHz broadband emitter and four regularly spaced receivers, was used to measure the axial velocity of ultrasound in the Achilles tendon of six healthy young adults (mean ± SD; age 26 ± 5 years; height 1.78 ± 0.11 m; weight 79.8 ± 13.6 kg) during steady-state unilateral hopping (2.5 Hz) on a piezoelectric force plate. Vertical ground reaction force and lower limb joint kinematics were simultaneously recorded. The potential sensitivity of the technique was further explored in subset of healthy participants (*n* = 3) that hopped at a slower rate (1.8 Hz) and a patient who had undergone Achilles tendon rupture-repair (2.5 Hz). Reliability was estimated using the mean-within subject coefficient of variation calculated at each point during the ground-contact phase of hopping, while cross-correlations were used to explore the coordination between lower limb kinematics ground reaction forces and ultrasound velocity in the Achilles tendon. Axial velocity of ultrasound in the Achilles tendon was highly reproducible during hopping, with the mean within-subject coefficient of variation ranging between 0.1 and 2.0% across participants. Ultrasound velocity decreased immediately following touch down (−19 ± 13 ms^–1^), before increasing by 197 ± 81 ms^–1^, on average, to peak at 2230 ± 87 ms^–1^ at 67 ± 3% of ground contact phase in healthy participants. Cross-correlation analysis revealed that ultrasound velocity in the Achilles tendon during hopping was strongly associated with knee (mean *r* = 0.98, range 0.95–1.00) rather than ankle (mean *r* = 0.67, range 0.35–0.79) joint motion. Ultrasound velocity was sensitive to changes in hopping frequency in healthy adults and in the surgically repaired Achilles tendon was characterized by a similar peak velocity (2283 ± 13 ms^–1^) but the change in ultrasound velocity (447 ± 21 ms^–1^) was approximately two fold that of healthy participants (197 ± 81 ms^–1^). Although further research is required, the technique can be used to reliably monitor ultrasound velocity in the Achilles tendon during hopping, can detect changes in the instantaneous elastic modulus of tendon with variation in hopping frequency and tendon pathology and ultimately may provide further insights into the stretch-shortening cycle and aid clinical decision concerning tendon rehabilitation protocols and readiness to return to sport.

## Introduction

Hopping capitalizes on the strain energy storage-recovery mechanism associated with the stretch-shortening cycle (Cavagna, 1977) and is emerging as an important component of progressive rehabilitation protocols in Achilles tendon injury and a determinant of readiness to return to sport following anterior cruciate ligament reconstruction (Noyes et al., 1991; Sancho et al., 2019; Silbernagel et al., 2020). The stretch-shortening cycle is characterized by pre-activation of the muscle followed by lengthening of the musculotendinous unit while the muscle remains activated and subsequent shortening of the musculotendinous unit, which is thought to make use of the elastic properties of tendon and optimize locomotor efficiency (Bobbert and Casius, 2011; Taube et al., 2012). This mechanism is dependent, in part, on the material stiffness (i.e., elastic modulus) of the posterior ankle tendons, and allows optimal function of the posterior muscles of the leg (Lichtwark and Wilson, 2005).

To date, measures of the elastic modulus of the Achilles tendon during hopping have typically been derived secondarily from estimates of tendon displacement and loading using B-mode ultrasound and indirect techniques, including inverse dynamics, and electromyography-to-force processing (Lichtwark and Wilson, 2005; Bogey and Barnes, 2017). Such indirect approaches are based on estimates of joint centers and tendon moment arms and require assumptions regarding the contribution of agonist and antagonist muscles to the net ankle joint moment, which may result in overestimation of tendon loads by as much as 50% compared to direct measures (Gregor et al., 1991; Fukashiro et al., 1993) and tendon work loops (hysteresis) that are physiologically implausible (Zelik and Franz, 2017).

While alternative approaches have been used to quantify soft tissue properties or loading *in vivo*, such as mechanical vibration and elastography (Martin et al., 2018; Corrigan et al., 2019; Keuler et al., 2019), transmission-mode ultrasound is emerging as potentially useful quantitative technique for the measurement of the mechanical properties of soft tissues such as tendon and ligament during activities of daily living (Miles et al., 1996; Kuo et al., 2001; Pourcelot et al., 2005a). The axial transmission velocity of ultrasound in tendon (V) is dependent on its instantaneous elastic modulus (E, material stiffness) and mass density (ρ) and, in tendon, is governed by the classic Newtonian–Laplace equation with adjustment for Poisson’s effects (ν) in elastic media (Miles et al., 1996; Pourcelot et al., 2005a; Crevier-Denoix et al., 2009; Vergari et al., 2012);

(Equation 1) $$V=\sqrt{\frac{E}{\rho }\,\frac{\left(1-\nu \right)}{\left(1+\nu \right)\,\left(1-2\,\nu \right)}}$$

Animal studies have confirmed that tensile stress applied to the tendon results in a nonlinear though monotonic increase in ultrasound velocity (Kuo et al., 2001; Pourcelot et al., 2005a; Crevier-Denoix et al., 2009). Hence, while the variation in the transmission speed of ultrasound with the application of mechanical load to tendon represents a limitation of most sonographic approaches (Ooi et al., 2014), ultrasound transmission techniques take advantage of this relationship, along with the nonlinear properties of tendon, to quantify the instantaneous elastic modulus of superficial human tendon *in vivo* under physiological loading conditions (Wearing et al., 2013, 2014; Wulf et al., 2015). Recently, the technique has been successfully applied to quantify tendon biomechanics during both static and dynamic activities of daily living such as walking and rising from a chair (Pourcelot et al., 2005b; Wearing et al., 2014, 2016) and has been shown to be sufficiently sensitive to detect changes in the instantaneous elastic modulus of Achilles tendon with footwear (Wearing et al., 2014), footstrike pattern (Wearing et al., 2019) and tendon pathology (Wulf et al., 2018). Although the technique has also been shown to be sufficiently sensitive to detect changes in the instantaneous elastic modulus of Achilles tendon with changes in walking speed (Brauner et al., 2017), there is debate as to whether the Achilles tendon normally operates within the elastic “toe” region under conditions of physiological loading or the “linear” elastic region, where Young’s elastic modulus is traditionally measured *in vitro* and ultrasound transmission velocity would approach a constant value (Maganaris and Paul, 1999; Biewener and Roberts, 2000). This is further compounded by direct measures of physiological loading in the Achilles tendon during submaximal hopping in which peak loads were found, rather unexpectedly, to be approximately twice those observed walking (Komi et al., 1992) and jumping (Fukashiro et al., 1995). Consequently, it remains unknown if the technique is suitable for individualized measurement of human Achilles tendon during hopping.

The primary aim of the current study, therefore, was to investigate the feasibility and reliability of transmission mode ultrasound in quantifying the instantaneous modulus of elasticity of human Achilles tendon during repetitive submaximal hopping. As a secondary aim, the coordination patterns between lower limb kinematics and ultrasound velocity profiles in the Achilles tendon were also explored. Finally, to further study the potential clinical utility and sensitivity of the technique, changes in peak ultrasound transmission velocity in the Achilles tendon were examined in a subgroup of participants hopping at a slower frequency and in a patient with a rupture-repaired Achilles tendon. It was anticipated that, as a surrogate measure of the instantaneous elastic modulus of the Achilles tendon, ultrasound velocity in the tendon would be lower when hopping at a faster rate, corresponding with a lower ankle joint moment (Otsuka et al., 2018), and also lower in rupture-repaired Achilles tendon as a consequence of a lower material stiffness and impaired ankle plantarflexion strength (Oda et al., 2017).

## Materials and Methods

### Participants

A convenience sample of six healthy young adults (three males, three females) participated in the study. The mean (±SD) age, height, and weight of healthy participants was 26 ± 5 years, 1.78 ± 0.11 m, and 79.8 ± 13.6 kg, respectively. In order to further explore the potential of transmission-mode ultrasound to evaluate the stretch shortening cycle, a female patient (age, 24 years; height, 1.76 m and weight, 54.0 kg) who had undergone unilateral surgical repair for rupture of the Achilles tendon was also evaluated. The participant was 24 months post-operation, pain free and had successfully returned to sport and normal activities of daily living and was individually matched to a healthy female participant to within five years of age and five kilograms. All participants were recreationally active, and regularly competed in structured sport (4 h per week). No participant reported a medical history of diabetes, familial hypercholesterolemia, inflammatory arthritis, or neuromuscular disease or a prior history of chronic Achilles tendon pain. All participants attended the laboratory on a single occasion for testing and provided written informed consent to the procedures of the study, which received approval from the University Human Research Ethics Committee.

### Equipment

**FIGURE 1:**
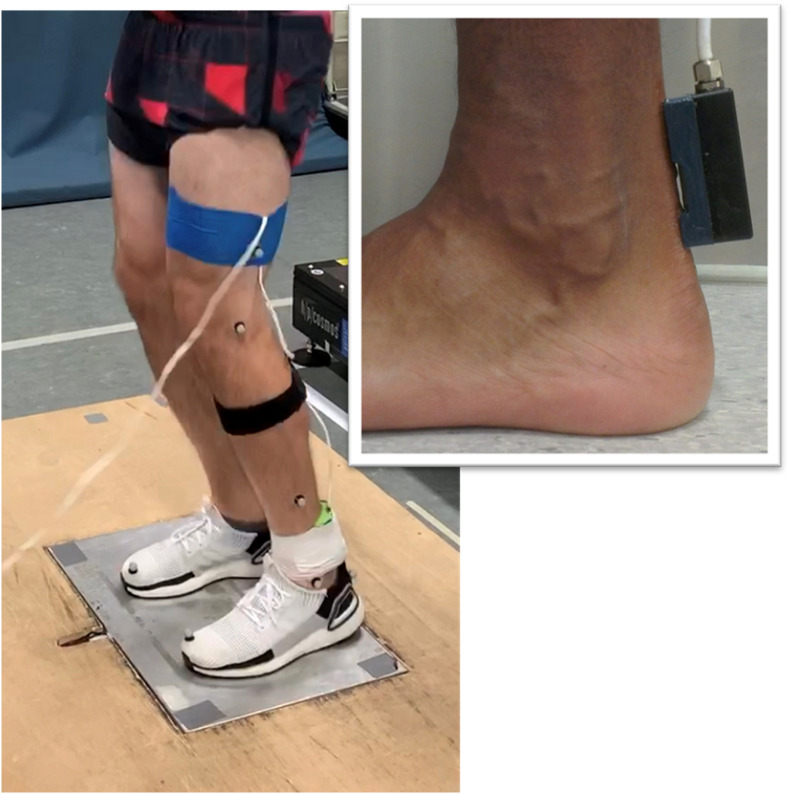
Illustration of the experimental setup. The custom-built ultrasound probe was positioned over the posterior Achilles tendon (insert).

Axial transmission velocity of ultrasound was measured in the Achilles tendon using a custom-built ultrasonic device described previously (Wearing et al., 2014, 2016). The device incorporated a five-element ultrasound probe consisting of a 1 MHz broadband pulse emitter and four regularly spaced receivers. The skin overlying the posterior Achilles tendon was prepared and cleaned using standard alcohol abrading methods (Cram and Rommen, 1989). The probe was positioned over the midline of the posterior aspect of the Achilles tendon during quiet bipedal stance, with the emitter located 1-cm superior to the calcaneus (Figure 1). Adhesive coupling medium and elasticized bandage were used to ensure the probe was maintained in close contact with the skin. Received ultrasonic signals were digitized at 20 MHz and the time of flight of the first arriving transient between receivers was estimated using the first-zero crossing criterion (Camus et al., 2000; Bossy et al., 2002). Average transmission velocity was subsequently calculated given the known distance between receivers (7.5 mm) and the measured time of flight. Measurement precision for axial transmission velocity of ultrasound is better than 3 ms^–1^ (Crevier-Denoix et al., 2009).

A 3D motion analysis system (Vicon Nexus 2.6, Vicon Motion Systems Ltd., Oxford, United Kingdom) was used to simultaneously record ankle and knee kinematics during the hopping task. The system consisted of eight high-resolution infra-red cameras (T10-series) that captured the kinematics of 16 reflective markers (14 mm diameter), positioned on standard anatomical landmarks of the hip, thigh, shank, and foot in accordance with Vicon’s Plug-in Gait Lower Body model (Kadaba et al., 1990; Davis et al., 1991). Sagittal plane motion of the knee and ankle joints during hopping were sampled at a rate of 200 Hz. Vertical ground reaction forces were also measured using a piezoelectric force platform (Kistler 9655, Kistler Instrument Corp., Amherst, NY, United States, natural frequency ∼600 Hz) sampling at 1000 Hz. Kinematic, kinetic and ultrasound data were collected synchronously using a common trigger.

### Protocol

A modified version of the repetitive hopping protocol outlined by Brauner et al. (2014) was employed. Prior to testing all participants were afforded a 5-min warm up in which they familiarized themselves with the sub-maximal hopping exercise. The protocol required participants to perform 20 s of single-limb hopping. Initially, participants stood erect for approximately 3 s, allowing a static calibration of lower extremity joint centers and coordinate systems, and then commenced hopping in place on the left limb until instructed to stop. A digital metronome (MA-30, KORG Inc., Japan) was used to set a hopping frequency of 2.5 Hz for all participants. To avoid the potential influence of arm movement, participants performed the hopping task with their hands laterally positioned on their waist. The hopping frequency was specifically selected, as the human leg is known to behave as an elastic spring at hopping rates above 2.2 Hz (Farley et al., 1991). To further explore the effect of hoping rate on ultrasound velocity measurements, three participants consented to repeat the hopping task at a slower rate (1.8 Hz) task.

### Data Reduction and Statistical Analysis

All data were low-pass filtered using a fourth-order, zero-lag Butterworth filter with a cut-off frequency of 30 Hz as determined by residual analysis. Data from the central ten steady-state hopping cycles over the 20 s period were selected for each participant for subsequent analysis. Each hopping cycle was identified based on vertical ground reaction force signals and resampled to 100 points per cycle. Peak vertical ground reaction force and peak loading rates for each hop cycle were subsequently determined (McSweeney et al., 2019). Sagittal ankle and knee angles and external moments during each hop cycle were calculated using standard “bottom-up” inverse dynamics via Plug-in-Gait. Maximum and minimum ultrasound velocity and sagittal ankle and knee angles were also identified. To allow direct comparison to previous studies, leg stiffness (K_dyn_) was defined as the instantaneous ratio of ground reaction force (F_G_) to the vertical displacement of the center of mass (ΔL) as given by;

(Equation 2) $${\text{K}}_{\text{dyn}}\;(\text{t})\;=\;{\text{F}}_{\text{G}}(\text{t})/\text{ΔL(t)}$$

where t equals time and the vertical displacement of the virtual hip joint center was used to describe the center of mass (Riese and Seyfarth, 2012). Importantly, the definition is equal to that cited by Farley and Morgenroth (1999) for the instant of maximum displacement of the center of mass.

All data were evaluated for normality using the Shapiro–Wilk test (IBM-SPSS statistical software package, Version 21 for Windows, IBM Corp. Armonk, NY, United States). As variables were determined to be normally distributed, means and standard deviations have been used as summary statistics. The within-subject coefficient of variation (SD/mean) for each participant was calculated at each point of the ground contact phase of all 10 hoping cycles and the mean was used as an estimate of intra-test (within-subject) variability. To visually compare data between participants, ensemble averages and standard deviations were computed. The standard deviation of mean within-subject data (i.e., mean across ten hopping cycles) was later used to estimate between-subject variability. The coordination between ultrasound velocity profiles in the Achilles tendon and lower limb kinematics and ground reaction forces were explored using cross-correlations (Winter and Patla, 1997; Mullineaux et al., 2001; Nelson-Wong et al., 2009). Absolute differences in mean peak ultrasound values that exceeded the minimum detectable change were used to compare the individual data of participants hopping at a slower rate and in the participant with a history of Achilles tendon rupture. The minimum detectable change was computed from the SEM using the equation described by Weir (2005).

## Results

**FIGURE 2:**
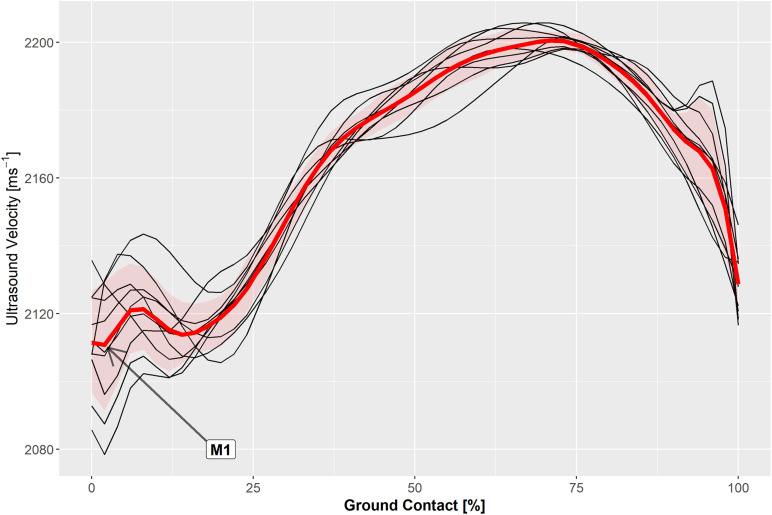
Axial velocity of ultrasound measured in the Achilles tendon during the ground contact phase of ten hop cycles (black traces) and the ensemble average (red trace) in a representative participant.

**FIGURE 3:**
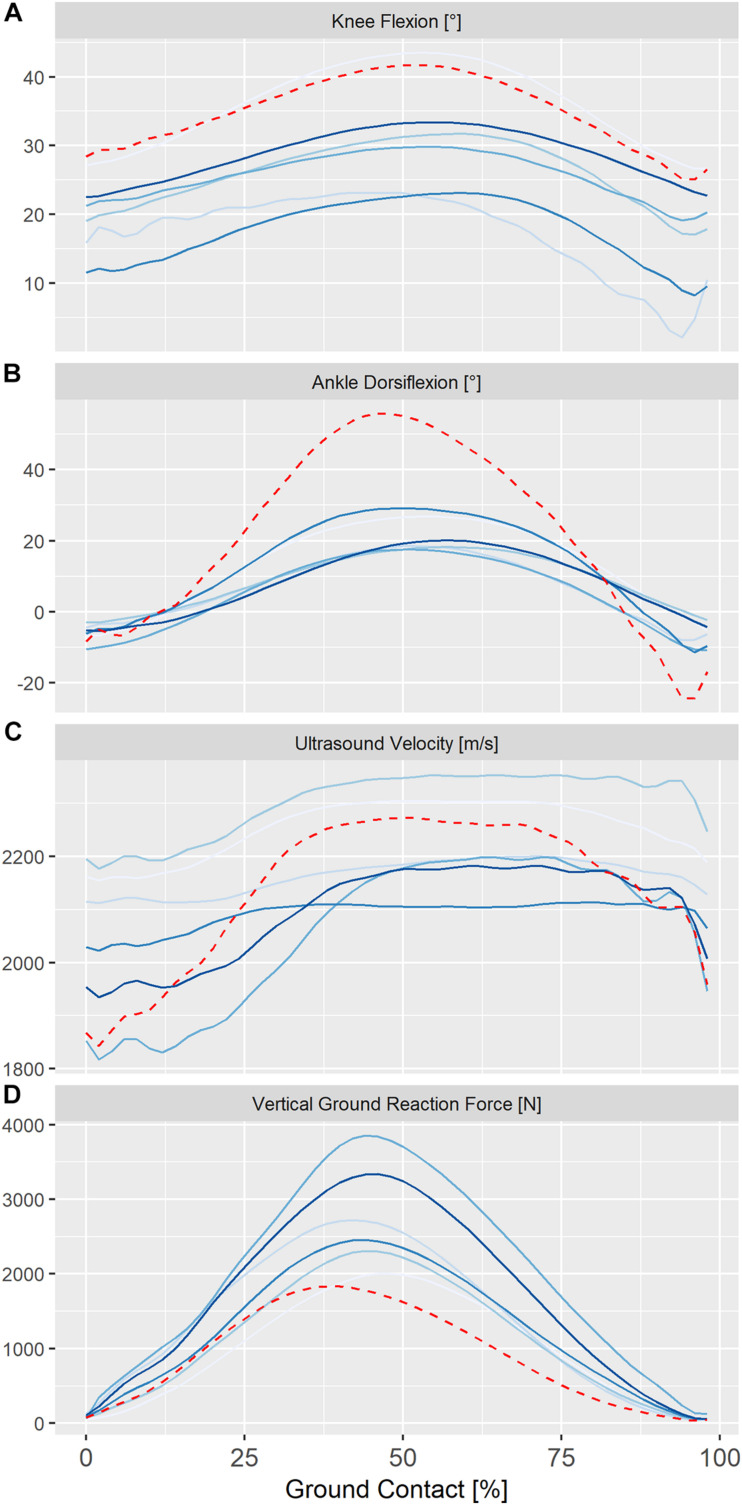
Individual ensemble histories for knee kinematics **(A)** ankle kinematics **(B)**, ultrasound velocity in the Achilles tendon **(C)** and vertical ground reaction forces **(D)** during the ground contact phase of steady-state hopping in six healthy adults (solid lines) and in a single adult, 24 months post-surgical repair (dashed line). Positive values for sagittal knee and ankle kinematics represent flexion and dorsiflexion, respectively.

Measured hopping frequencies were 2.51 ± 0.02 Hz for all participants and 1.86 ± 0.02 Hz for the three participants that repeated the exercise at a slower rate and coinciding with a hopping height of 3.6 to 4.1 cm. The ultrasound velocity profile in the Achilles tendon was highly reproducible (Figure 2) and was typically characterized by a local minimum (M1) and maximum (P1) during the contact phase of unilateral steady-state hopping (Figure 3). Variability in ultrasound velocity in the Achilles tendon tended to be greatest during initial touchdown (1.1%) and lowest at around the time of peak vertical ground reaction force, where the mean within-subject coefficient of variation for ultrasound velocity in the tendon was, on average, less than 0.3%. The mean within-subject coefficient of variation for ultrasound velocity over the entire ground contact phase of the hopping cycle was 0.9% and ranged between 0.1 and 2.0% across all participants.

Ensemble averages for knee and ankle kinematics and axial velocity of ultrasound in the Achilles tendon for all participants are shown in Figure 3. On average, peak ultrasound velocity in the Achilles tendon of healthy participants decreased by 19 ± 13 ms^–1^ immediately following touch down at 4 ± 2% of ground contact, before increasing to peak at 2230 ± 87 ms^–1^ at 67 ± 3% of ground contact phase (Table 1). The between-subject variability, as defined by the mean ensemble standard deviation (100 ± 21 ms^–1^), was approximately five-fold greater than the intra-test (within-subject) variability. Cross-correlation analysis revealed that ultrasound velocity in the Achilles tendon during hopping was strongly associated with knee (mean *r* = 0.98, range 0.95–1.00) rather than ankle (mean *r* = 0.67, range 0.35–0.79) joint motion in the six healthy participants (Table 2).

**TABLE 1:** Mean (SD) kinematic, kinetic, and ultrasound parameters during the contact phase of hopping in healthy adults (*n* = 6).

	**Contact**	**M1**	**P1**	**Toe off**	**Time to M1 (%)^†^**	**Time to Peak (%)^†^**
Knee angle (°)	20 (5)	–	31 (8)	18 (7)	–	54 (6)
Ankle angle (°)	−6 (3)	–	22 (5)	−6 (3)	–	54 (3)
Ultrasound velocity (ms^–1^)	2051 (131)	2032 (141)	2230 (87)	2097 (113)	4 (2)	67 (3)
Vertical ground reaction force (BW)		–	3.6 (0.5)		–	45 (2)
Leg stiffness (kNm^–1^)		–	32 (12)		–	21 (13)

*^†^Expressed as a percentage of the duration of ground contact; BW, normalized to bodyweight. Positive knee and ankle angles reflect flexion and dorsiflexion, respectively.*

**TABLE 2:** Cross correlation (range) and lag^†^ of kinematic, kinetic and ultrasound parameters during hopping.

	**Ultrasound velocity**	**Knee angle**	**Ankle angle**	**Vertical ground reaction force**
Knee angle	0.98 (0.95 to 1.00)			
Lag (%)	0 (0 to 0)			
Ankle angle	0.67 (0.35 to 0.79)	0.73 (0.36 to 0.86)		
Lag (%)	3 (−14 to 28)	9 (−4 to 20)		
Vertical ground reaction force	0.85 (0.83 to 0.87)	0.91 (0.89 to 0.95)	0.90 (0.62 to 0.99)	
Lag (%)	4 (0 to 8)	3 (^–^2 to 6)	8 (6 to 10)	
Leg stiffness	0.91 (0.88 to 0.93)	0.95 (0.92 to 0.96)	0.83 (0.53 to 0.94)	0.96 (0.94 to 0.98)
Lag (%)	2 (0 to 4)	6 (0 to 12)	19 (16 to 22)	10 (6 to 16)

*^†^Expressed as a percentage of the duration of ground contact.*

**FIGURE 4:**
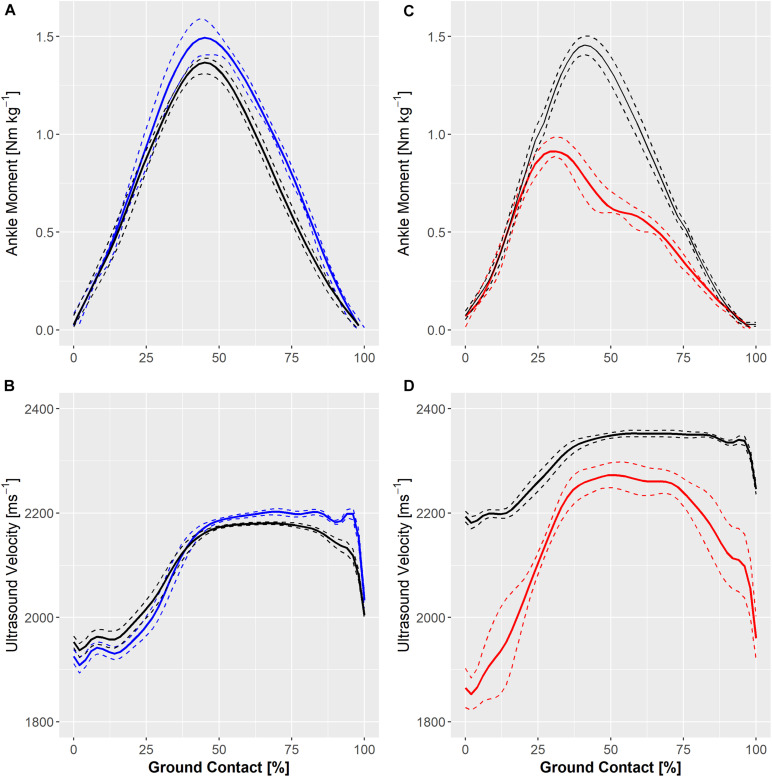
Individual ensemble histories for external ankle moments (upper panels) and ultrasound velocity in the Achilles tendon (lower panels) of a healthy adult during hopping at 1.8 Hz (solid blue line) and 2.5 Hz **(A,B)** and in an adult with a surgically repaired Achilles tendon (solid red line) and a matched healthy control hopping at 2.5 Hz **(C,D)**. Standard deviations are represented by dashed lines.

Ensemble ankle moments and ultrasound velocity in the Achilles tendon at slow (1.8 Hz) and fast (2.5 Hz) hopping rates in a healthy individual are shown in Figure 4. Hopping at a faster rate resulted in a qualitative reduction in knee and ankle movement and an increase in peak ground reaction force and ultrasound velocity in the Achilles tendon of two healthy participants but a lower peak ultrasound velocity and external ankle moment in the third participant (Figure 4A). Figure 4 also demonstrates ultrasound velocity and ankle moments during hopping (2.5 Hz) in the participant that had undergone operative repair of the Achilles tendon relative to that of an age-, weight-, and sex-matched healthy control. Hopping in the surgically repaired Achilles tendon was characterized by markedly lower ankle moment (Figure 4B). Although peak ultrasound velocity (2283 ± 13 ms^–1^) was comparable to healthy participants hopping at the same frequency, the change in ultrasound velocity from touch down to peak velocity (447 ± 21 ms^–1^) was approximately two fold that of healthy participants on average (197 ± 81).

## Discussion

Measurement of ultrasound velocity was well tolerated by participants in the current study and without technical difficulty. The ultrasound velocity profile in the Achilles tendon was characterized by a local minimum and maximum during the contact phase of unilateral steady-state hopping. Axial velocity of ultrasound in the Achilles tendon was highly reproducible between hopping cycles, with a mean intra-test (within-subject) coefficient of variation of less than 1% over the entire ground contact phase. Between-subject variability in ultrasound velocity, however, was about five times higher than within-subject variability. The greater variability between subjects presumably reflects the various hopping movement patterns or strategies adopted by individuals during hopping (Chang et al., 2008). However, given that animal models have shown the coefficient of variation in mass density of different tendons to be in the order of 2.8% (Vergari et al., 2012), the variability in ultrasound velocity observed between healthy participants in the current study may also reflect differences in tendon cross-sectional area and density between participants. Nonetheless, the maximum and minimum ultrasound velocities measured in the Achilles tendon during the stretch-shortening cycle in hopping in this study (approximately 2000–2230 ms^–1^) are somewhat higher than those reported previously in healthy adults (approximately 1850–2050 ms^–1^) during walking at preferred speed (Pourcelot et al., 2005a,b; Wearing et al., 2014)). Assuming a mass density of approximately 1100 kg/m^3^ (Vergari et al., 2012), and a Poisson’s ratio of around 0.46 (Thorpe et al., 2014), the minimum and maximum ultrasound velocity observed in the Achilles tendon during the stretch-shortening cycle of hopping in the current study equate to an elastic modulus of 0.98 and 1.2 GPa, respectively, which is comparable to that estimated for human cadaveric tendon (819 ± 208 MPa) during quasistatic tensile testing (Wren et al., 2001).

Peak vertical ground reaction force, joint kinematics and estimates of leg stiffness in the current study were consistent with those reported for hopping in healthy adults at similar frequencies (Farley et al., 1991). Leg stiffness during hopping at frequencies above 2.2 Hz have previously been suggested to predominantly reflect ankle joint stiffness (Farley and Morgenroth, 1999; Hobara et al., 2011). Although ankle joint stiffness may be regulated through a number of mechanisms, passive tissue properties, stretch reflexes, and muscle activation are all thought to contribute to physiological joint stiffness (Weiss et al., 1986a,b, 1988). In the absence of the ankle joint approaching the limits of its range of motion, simultaneous co-activation of agonist-antagonist muscles has been suggested to be a prime method for increasing the mechanical stiffness of the ankle joint, without necessarily affecting the angle or net torque (Wind and Rouse, 2020). Hence, co-activation of antognist muscle groups, as has been previously reported with jumping (Arai et al., 2013), may be one explanation for the elevated velocity of ultrasound in Achilles tendon during the propulsive phase of hopping; despite a concomittent reduction in vertical ground reaction force. Interestingly, ultrasound velocity in the Achilles tendon was more closely correlated with knee flexion than ankle movement in the current study. Although this observation highlights the challenge associated with deconvoluting the contribution of agonist and antagonist muscles to the net joint moment, especially of biarticular muscles such as the triceps surae, it also suggests that, at least in this study, the ultrasound velocity profile developed within the triceps surae muscle unit was related more to knee flexion than ankle movement during hopping in healthy adults. This observation is consistent with synergistic interaction of the ankle and knee joints proposed by João et al. (2014), in which the knee extensors were considered critical to the vertical acceleration of the center of mass, while the ankle plantarflexors acted to stabilize the foot and ankle.

The ultrasound transmission technique also appears sufficiently sensitive to demonstrate individual responses to changes in hopping frequency and to characterize deficits in dynamic tendon function in a participant with a history of Achilles tendon rupture repair. It is noteworthy that, in the latter case, that the participant was asymptomatic, reported good function and had returned to sport. Ankle joint dorsiflexion but not knee flexion was markedly increased during hopping in the rupture-repaired tendon. Increased ankle dorsiflexion during hopping is a well-known complication following Achilles tendon rupture repair (Tengman and Riad, 2013). Despite having the lowest external ankle joint moment of any participant, peak ultrasound velocity in the repaired Achilles tendon was comparable to that of healthy participants (2120–2358 ms^–1^). Interestingly, the change in ultrasound velocity during hopping in the surgically repaired tendon was twice that of healthy tendon, suggesting that the muscle-tendon unit of rupture-repaired Achilles tendon may be more compliant than that of healthy tendon. While this initial finding is consistent with previous research demonstrating that repaired Achilles tendon is longer (Rosso et al., 2013), has a lower material stiffness (Wulf et al., 2018) and is associated with lower ankle plantarflexion strength than healthy tendon (Horstmann et al., 2012), further prospective research in surgically repaired, contralateral and healthy Achilles tendon under both static and dynamic loading conditions is warranted to further elucidate the effect of tendon repair on tendon function.

Although the change in ultrasound transmission velocity in tendon over the hop cycle provides an indication of the stress developed in the muscle-tendon unit as a whole, it should be noted that tendon stress is influenced by tendon cross-sectional area. As the Achilles is a conjoined tendon of the soleus and medial and lateral gastrocnemius muscles, differential muscle activity and loading within the tendon may also occur (Arndt et al., 1998). Hence, whether the ultrasound velocity pattern observed in the current study is applicable to the entire tendon structure is unknown. Similarly, variations in ultrasound transmission velocity during hopping are influenced by both active (muscle) and passive components of loading. Hence, the role of active and passive loading mechanisms could not be readily delineated. While future studies may benefit from evaluating tendon properties in the absence of external loads and under passive loading conditions, it is recognized that *in vivo* measurement of tendon slack is not straightforward (Hug et al., 2013). Moreover, the transmission of ultrasound waves in tendon is also influenced by factors such as the density and temperature of the tissue (Miles et al., 1996). Although it may reasonably be assumed that, for a given individual, tendon density and temperature remained stable during the short-term, steady-state hopping conditions tested in the current study, repeated cyclic loading of tendon has been estimated to induce considerable thermal effects in equine tendon when applied over an extended period (Wilson and Goodship, 1994). Marked variation in such factors may then make comparisons between-individuals more difficult. In addition, the technique is only suitable for evaluating the biomechanics of relatively superficial tendons. It should also be noted that this study employed conventional motion analysis to model the foot as a rigid segment to estimate ankle joint movement, which undoubtedly overestimates peak osseous ankle movement. The range of ankle joint movement during hopping in the current study, however, is consistent with that reported for the tibiotalar joint when bone pins and dyanamic radiographic approaches were used to track osseous movement (Pitcairn et al., 2020).

Nonetheless, the limited data presented in this study have shown that transmission-mode ultrasound can be reliably used to quantify tendon biomechanics during hopping in healthy and injured Achilles tendons. As ultrasound velocity is directly related to the instantaneous elastic modulus of the Achilles tendon, and the change in velocity reflects the change in applied stress to the tendon, the technique also has the potential to provide new insights into muscle-tendon unit function during the stretch-shortening cycle, particularly if combined with synchronous measures of muscle activity and fascicle behavior. Hence, the technique has application for research concerning the neuromuscular control of movement, the role of elasticity in hopping and neuromuscular adaptation and pathology. Moreover, the ultrasound technique shows promise for near real-time clinical monitoring of Achilles tendon properties during hopping, which is emerging as an important component of progressive rehabilitation protocols and a key clinical determinant of the readiness to return to sport.

## Data Availability Statement

Summary data supporting the conclusions of this article will be made available by the authors, without undue reservation.

## Ethics Statement

This study was reviewed and approved by the University of Applied Health Sciences Rhein-Neckar. The participants provided their written informed consent to participate in this study.

## Author Contributions

LK, SW, TB, and TP designed the experiment. LK and TP collected the data. SW, TB, and TP performed the statistical analysis. SW, TB, and TH interpreted the results. SW wrote the first draft of the manuscript. SW and TB prepared the figures. All authors contributed to the manuscript revision, read, and approved the submitted version.

## Conflict of Interest

The authors declare that the research was conducted in the absence of any commercial or financial relationships that could be construed as a potential conflict of interest.
